# Working Memory Modulates Glutamate Levels in the Dorsolateral Prefrontal Cortex during ^1^H fMRS

**DOI:** 10.3389/fpsyt.2018.00066

**Published:** 2018-03-06

**Authors:** Eric A. Woodcock, Chaitali Anand, Dalal Khatib, Vaibhav A. Diwadkar, Jeffrey A. Stanley

**Affiliations:** ^1^Brain Imaging Research Division, Department of Psychiatry and Behavioral Neurosciences, Wayne State University School of Medicine, Detroit, MI, United States

**Keywords:** magnetic resonance spectroscopy, glutamate, working memory, excitatory neurotransmission, metabolism, dorsolateral prefrontal cortex, neuroimaging

## Abstract

Glutamate is involved in excitatory neurotransmission and metabolic processes related to brain function. Previous studies using proton functional magnetic resonance spectroscopy (^1^H fMRS) have demonstrated elevated cortical glutamate levels by 2–4% during visual and motor stimulation, relative to periods of no stimulation. Here, we extended this approach to working memory cognitive task performance, which has been consistently associated with dorsolateral prefrontal cortex (dlPFC) activation. Sixteen healthy adult volunteers completed a continuous visual fixation “rest” task followed by a letter 2-back working memory task during ^1^H fMRS acquisition of the left dlPFC, which encompassed Brodmann areas 45 and 46 over a 4.5-cm^3^ volume. Using a 100% automated fitting procedure integrated with LCModel, raw spectra were eddy current-, phase-, and shift-corrected prior to quantification resulting in a 32s temporal resolution or 8 averages per spectra. Task compliance was high (95 ± 11% correct) and the mean Cramer-Rao Lower Bound of glutamate was 6.9 ± 0.9%. Relative to continuous passive visual fixation, left dlPFC glutamate levels were significantly higher by 2.7% (0.32 mmol/kg wet weight) during letter 2-back performance. Elevated dlPFC glutamate levels reflect increased metabolic activity and excitatory neurotransmission driven by working memory-related cognitive demands. These results provide the first *in vivo* demonstration of elevated dlPFC glutamate levels during working memory.

## Introduction

**Figure 1:**
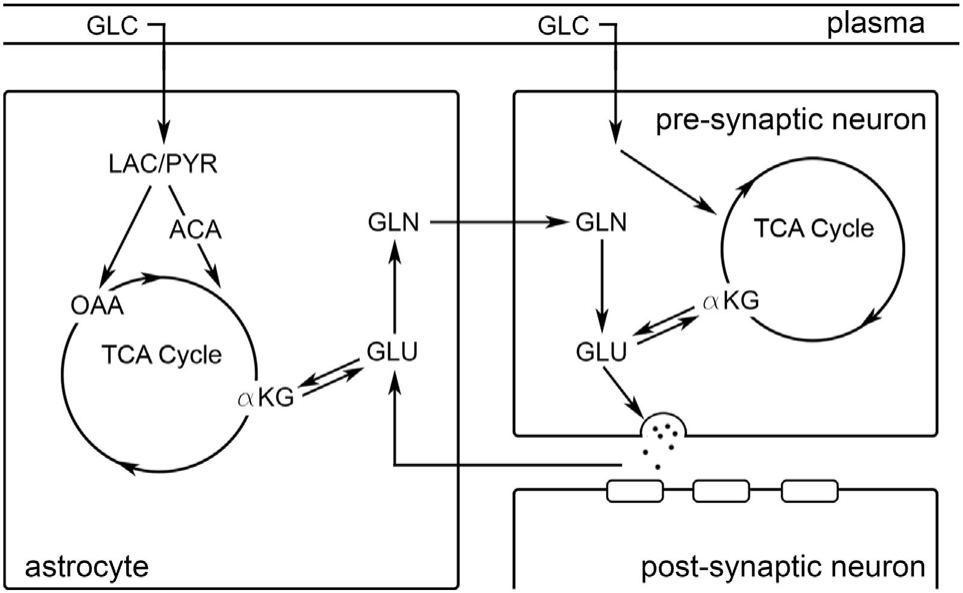
A schematic representation of the glutamatergic tripartite synapse is depicted. Black arrows illustrate relationships between molecular species. ACA, acetyl-CoA; αKG, α-ketoglutarate; GLC, glucose; GLN, glutamine; GLU, glutamate; LAC, lactate; OAA, oxaloacetate; PYR, pyruvate.

Glutamate is the most abundant neurotransmitter in the brain: ~10–12 mM (1). In addition to its role as the primary excitatory neurotransmitter, glutamate is also involved in the metabolic processes such as the tricarboxylic acid [TCA] cycle and is a neurochemical intermediate for other metabolites, such as GABA and glutamine (Figure 1) (2). Functional proton magnetic resonance spectroscopy (^1^H fMRS) facilitates exploration of *in vivo* glutamate dynamics in localized brain volumes in response to task-related stimulation (3). Seminal ^1^H fMRS research, conducted at 7 T, demonstrated visual stimulation increased glutamate levels by ~2–4% in the occipital lobe (4, 5). Subsequent ^1^H fMRS studies observed significant glutamate modulation throughout the brain: (1) occipital lobe during visual stimulation (6–10); (2) motor cortex during finger tapping (11); (3) anterior cingulate cortex during Stroop task performance (12–14); (4) hippocampus during associative memory (15); and (5) elsewhere (16–18). Extensive ^13^C MRS research demonstrates that glutamate involved in excitatory neurotransmission (glutamate–glutamine cycling) and neuronal oxidative metabolism of glucose exhibit a tight, nearly 1:1, stoichiometric relationship in rodents (19–24) and humans (25–30) [see review (2)]. Thus, task-induced glutamate modulation, as measured *via* ^1^H fMRS, is interpreted as *in vivo* biomarker of increased metabolic activity and excitatory neurotransmission. Indeed, numerous studies have demonstrated task-induced glutamate modulation is co-localized with blood oxygen-level dependent (BOLD) activation (5, 7, 9, 10, 31).

Working memory involves the neuronal representation of stimuli over a brief delay period (32). Electrophysiology research in non-human primates indicate that persistent neuronal spiking through feed-forward excitatory microcircuits in cortical layer III in the dorsolateral prefrontal cortex (dlPFC) is associated with the maintenance of location information during spatial working memory tasks (32). Follow-up pharmacological challenge studies illustrated the central role of glutamate binding post-synaptic N-methyl-d-aspartate (NMDA) receptors during spatial working memory (33). To date, only one human study has investigated the dynamic *in vivo* neurochemistry of working memory processes (34). Using ^1^H fMRS at 3 T, Michels and colleagues found GABA levels were elevated, relative to baseline, during the first block of a letter Sternberg working memory task (34). Interestingly, GABA levels decreased with each successive repetition of the task, despite no decrement in performance, which illustrated the dynamic nature of neurochemistry during working memory processes (34). The focus of that study was investigation of GABAergic dynamics and the authors did not report isolated glutamate levels (34). Thus, dynamic glutamate modulation during working memory processes in humans remains unclear.

Here, we used ^1^H fMRS to investigate glutamate levels in the dlPFC during working memory task performance. The letter 2-back task is a well-established working memory paradigm reliably associated with dlPFC activation (35). Consistent with the literature, we hypothesized that letter 2-back task performance would be associated with elevated *in vivo* glutamate levels, relative to passive visual fixation, in the left dlPFC.

## Materials and Methods

### Participants

The local Institutional Review Board approved all study procedures, which were conducted in accordance with the Declaration of Helsinki (1964). Male and female right-hand dominant volunteers between 18 and 30 years old without self-reported MR contraindications, psychiatric conditions, and not currently taking psychoactive medications were recruited locally. Eligible subjects provided informed consent and were compensated for their time. Sixteen participants, nine males and seven females, completed self-report measures including medication history, demographic questionnaire and contact information, a comprehensive MRI safety screen, and the MRI scan, which was approximately 60min.

### Experimental Tasks

**Figure 2:**
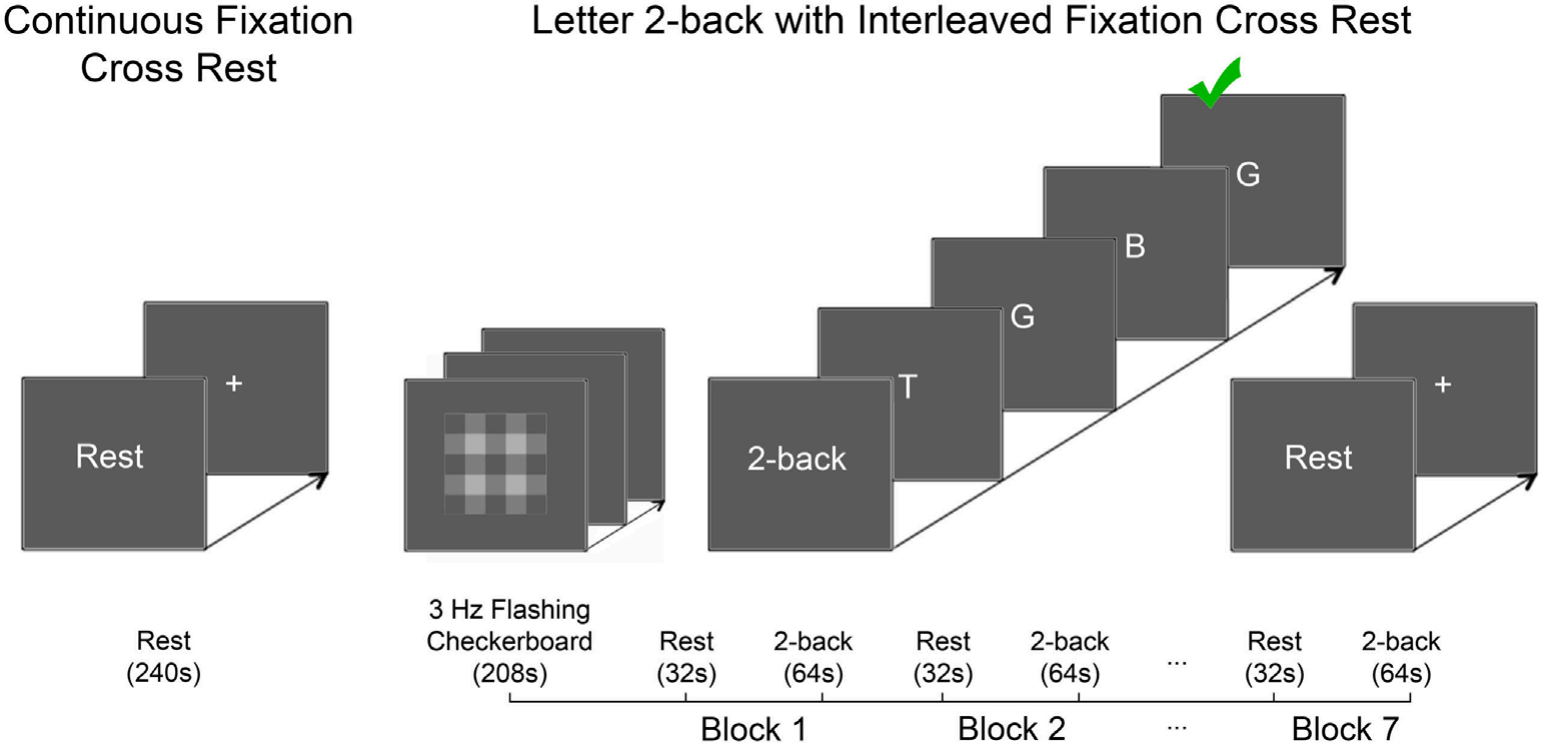
The experimental tasks are depicted. Left panel: the continuous passive visual fixation task consisted of instructions (“Rest”; 2s) and static fixation cross (centered on-screen; 238s). Right panel: the letter 2-back task consisted of two phases: variance minimization and alternating periods of letter 2-back interleaved with periods of passive visual fixation. The variance minimization phase consisted of a flashing grayscale checkerboard (centered on-screen; 3Hz) presented for 208s. Previous research in our laboratory demonstrated that the flashing checkerboard minimized glutamate variance better than alternative approaches (36). The working memory task consisted of seven interleaved repetitions of passive visual fixation [32s; 2s of instructions (“Rest”), 30s of static fixation cross centered on-screen] and letter 2-back [64s; 4s of instructions (“2-back”), 20 capitalized letters presented serially 3s/letter (60s total); each letter was presented on screen for 500ms followed by 2,500ms of blank screen].

Prior to the ^1^H fMRS scan, participants were verbally instructed how to perform each experimental task (Figure 2). Outside the MRI scanner, each participant practiced the letter 2-back task until deemed proficient by the experimenter. Inside the MRI scanner, participants completed the continuous passive visual fixation task, which consisted of a 2s “Rest” prompt, followed by static, continuous fixation-cross presentation for 238s. Next, participants completed the working memory task, which consisted of a 3Hz flashing grayscale checkerboard task for 208s followed by 7 blocks of alternating periods of passive visual fixation cross of 32s and letter 2-back of 64s. Each block of interleaved passive visual fixation was initiated on screen with a 2s “Rest” prompt prior to a static fixation cross for 30s totaling 32s. During each 2-back block, instruction of a 4s “2-back” prompt was followed by serial presentation of 20 capitalized letters out of which, 6 were randomly-assigned targets. Each letter was displayed for 500ms followed by 2,500ms of blank screen. Subjects indicated *via* button press with their right index finger, if the letter on the screen matched the letter presented two previously. For each task block, response accuracy was quantified as a percentage of correct responses out of 6 target letters. Participants were not provided feedback about response accuracy.

### Neuroimaging

All imaging was conducted on a 3T Siemens Verio system with a 32-channel receive-only volume head coil. All scans were completed in the morning between 9:00 and 11:30 a.m. The imaging protocol was identical for each subject. High resolution T_1_-weighted structural scans were collected using the 3D Magnetization Prepared Rapid Gradient Echo (MPRAGE) sequence with the following parameters: TR = 2.2s, TE = 3ms, TI = 799ms, flip angle = 13°, field-of-view (FOV) = 256mm × 256mm × 160mm, acquired matrix = 176 × 256 × 160, and pixel resolution 1mm × 1mm × 1mm. Prior to ^1^H fMRS acquisition, a region of the left dlPFC (25mm × 25mm × 25mm) larger than ^1^H fMRS voxel was shimmed to improve B_0_-field homogeneity [FASTESTMAP (37)]. ^1^H fMRS spectra were continuously acquired every 16s during each experimental task, which included 15 spectra for the continuous visual fixation and 55 spectra for the 2-back task. The acquisition parameters included the PRESS sequence with OVS and VAPOR for water suppression, TE = 23ms, TR = 4.0s, 4 averages per spectrum, bandwidth = 2kHz, 2,048 complex data points, and no apodization or zero-filling. A relatively short TE reduced the influence of J-evolution and diffusion, and the relatively long TR ensured minimal T_1_-weighted effect on the acquired signal. Fully relaxed water unsuppressed ^1^H fMRS spectra from the same voxel location and using a TR = 10s and 2 averages were acquired immediately after the continuous passive visual fixation and after the 2-back task, which was used for absolute quantification calculations.

### Voxel Placement

**Figure 3:**
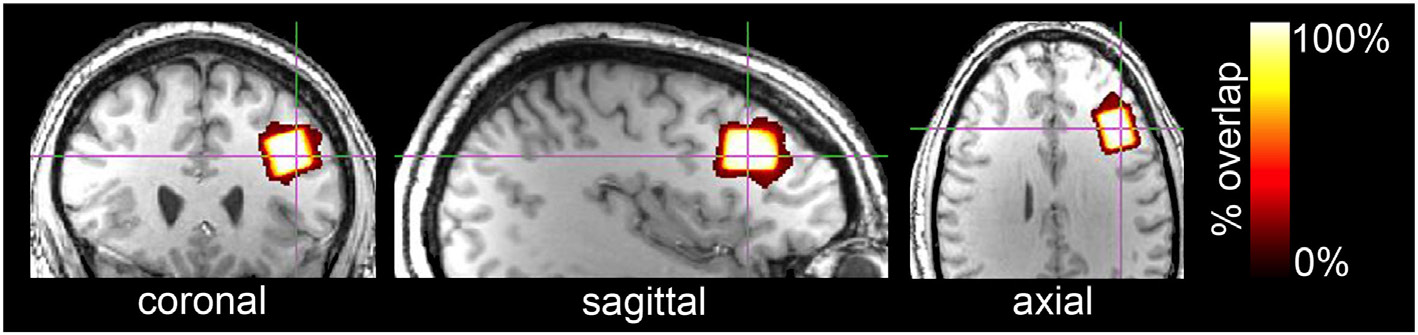
Orthonormal slices of voxel overlap across all subjects are depicted in a template brain. The voxel (15mm × 20mm × 15mm; 4.5cm^3^) was located in the left dlPFC and included Brodmann areas 45 and 46. 3D geometric voxel overlap is indicated by the red-to-white color gradient (white = complete voxel overlap across all subjects; orange-red = incomplete overlap across subjects). Voxel placement reliability across all subjects was excellent (89.9%).

^1^H fMRS spectra were acquired from the left dlPFC with a voxel dimension of 15mm × 20mm × 15mm or 4.5cm^3^, which encompassed Brodmann areas 45 and 46 (Figure 3). Voxel location, which was determined *a priori* and based on the results from a meta-analysis of 2-back fMRI studies (35), was determined on the MNI standard brain. From there, the automated voxel placement (AVP) method (38), which mapped the voxel location from template space to subject space, was used to prescribe 15 of the 16 participants’ voxel locations. AVP was not used for one subject due to experimenter error.

### Analysis Strategy

^1^H fMRS spectra were analyzed using LCModel, version 6.3, with a simulated basis set (39). The first spectrum acquired for the continuous passive visual fixation and letter 2-back tasks was not considered in analyses due to a potential partial T_1_ saturation effect (40). Post-processing and metabolite quantification steps were 100% automated. Eddy current effects were corrected using the unsuppressed water signal (41). T_1_-weighted structural images were B_1_-field corrected, the brain extracted, and segmented into partial volume maps of cerebrospinal fluid, gray and white matter using FreeSurfer and FSL tools (42, 43). Finally, tissue composition within the MRS voxel and appropriate correction factors, such as T_1_ and T_2_ relaxation, were used to calculate absolute glutamate levels (mmol/kg wet weight) (44). Consecutive phase- and shift-corrected spectra were averaged for the 2-back blocks, continuous and interleaved passive visual fixation conditions, which resulted in spectra with 8 averages and 32s temporal resolution. This resulted in bisecting the 64s 2-back blocks into a first- and second-half outcome measurement for each 2-back task block, which are denoted as “2-back-A” and “2-back-B,” respectively. Thus, each task phase included seven 32s outcome measurements. Rationale for this approach was twofold: (1) We used a classical block design, often employed in fMRI studies. Thus, to avoid signal-to-noise-ratio (SNR) and glutamate fit bias, we contrasted task phases using a common temporal resolution. (2) Exploratory analyses examined dynamic glutamate levels across task-repetitions, consistent with prior research (34).

Shapiro–Wilk test of normality and skewness/kurtosis statistics were used to evaluate variable distributions prior to outcome analyses. Whenever necessary, extreme values (≥1 SD from nearest value) were winsorized. The extreme value was replaced with the nearest value such that distributions were normalized with minimal influence on group mean. *A priori* hypotheses were evaluated *via* four one-way repeated measures analyses of variance (rmANOVA) that separately contrasted glutamate levels during 2-back-A and 2-back-B vs. interleaved passive visual fixation and continuous passive visual fixation. Power analyses conducted using G*Power (v3.0.10) indicated that 16 subjects were adequate to detect a small-to-moderate within-subject main effect (*f* ≥ 0.20) across 7 task repetitions at the recommended power (0.80) and α = 0.05 for autocorrelated measures (*r* = 0.70) (45, 46). Descriptive statistics were used to clarify significant main effects. In addition, four hypothesis-generating exploratory analyses were conducted. First, exploratory zero-order correlations were used to examine possible relationships between 2-back response accuracy and glutamate levels (uncorrected; *p* < 0.05). Second, one-way rmANOVAs evaluated possible temporal glutamate effects for each task phase (Bonferroni-corrected; *p* < 0.0125). Third, we evaluated other metabolites measured with Cramer Rao Lower Bound (CRLB%) < 20%, which included N-acetyl-aspartate (NAA), phosphocreatine plus creatine (PCr+Cr), glycerophosphocholine plus phosphocholine (GPC+PC), and *myo*-Inositol, to determine the neurochemical specificity of working memory task-related modulation. For each metabolite, two-way rmANOVAs evaluated 2-back-A and 2-back-B separately using a Bonferroni-correction (*p* < 0.00625). Fourth, LCModel fit characteristics, which included glutamate CRLB%, full-width half maximum (FWHM), and SNR, were evaluated separately for 2-back-A and 2-back-B using two-way rmANOVAs that were Bonferroni-corrected (*p* < 0.0083).

Voxel overlap was quantified using the “avp_overlap” script included in the AVP suite (38). Each subject’s voxel was coregistered to template space, and 3D geometric voxel overlap percentage reflecting placement accuracy and voxel overlap across all subjects reflecting reliability were calculated. Descriptive statistics are presented as mean ± 1 SD, unless otherwise noted. In all figures, error bars depict ± 1 SEM. Coefficient of variation percentage (CV%) was calculated to evaluate glutamate signal variability across time.

## Results

### Sample Characteristics

The sample consisted of 16 healthy college-educated participants not taking psychoactive medications. The mean age of the participants was 24 years old (± 3.4 years; range: 19–30 years), and the sample composition was 50% Caucasian or Asian and 56% male.

### Voxel Overlap

The voxel placement using AVP (38) was highly accurate with a mean 3D geometric overlap percentage relative to the template voxel of 92.3 ± 4.7% and reliable across participants with a shared voxel overlap of 89.9% (Figure 3). Mean voxel tissue composition was 36.8 ± 3.8% gray matter and 60.8 ± 4.5% white matter. The manual voxel placement of one participant’s voxel was less accurate to the template (77.7%) than AVP placed voxels (86.2–96.9%).

### Behavioral Data

**Figure 4:**
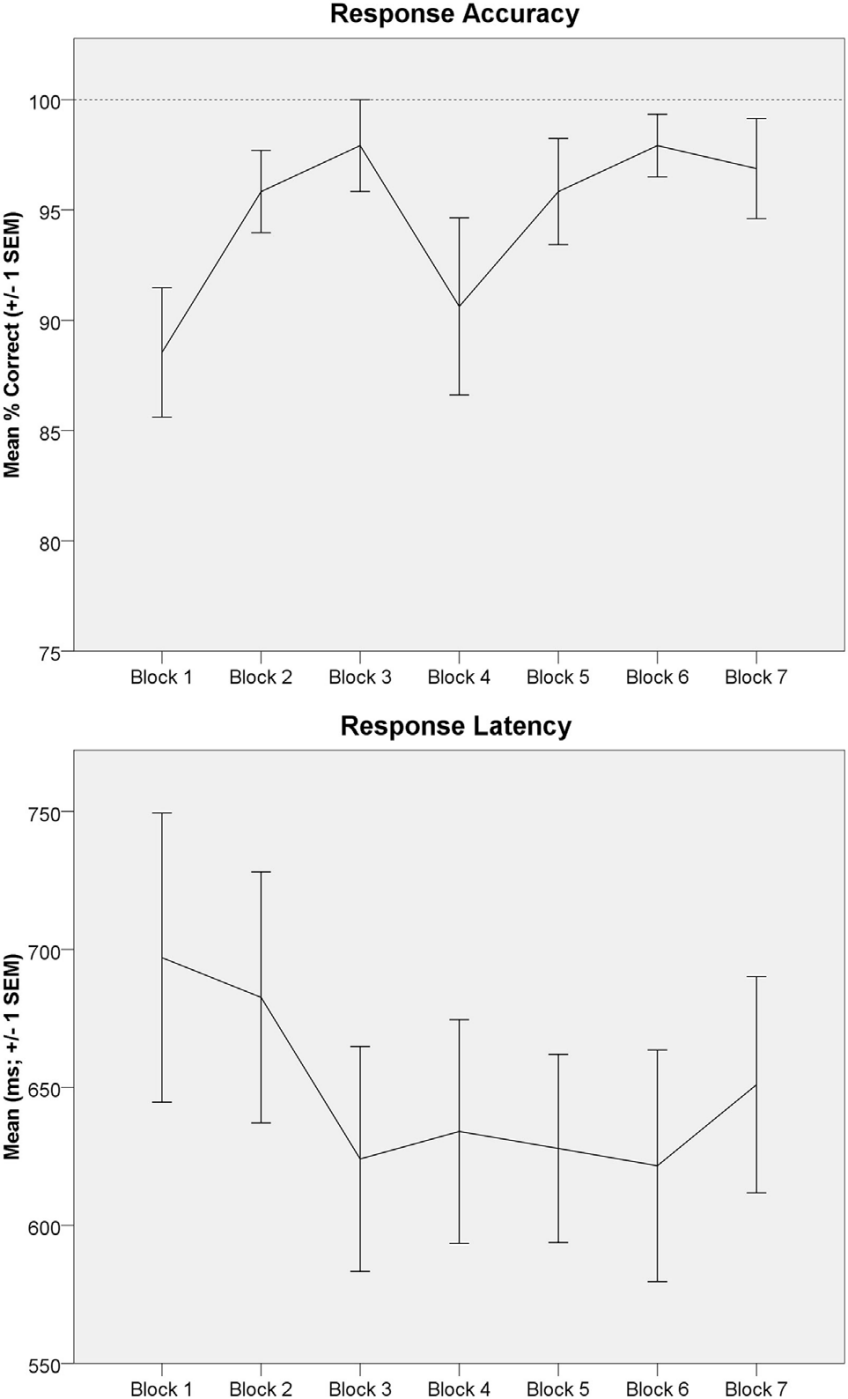
Letter 2-back behavioral data are depicted. Upper panel: letter 2-back response accuracy (mean percentage correct ± 1 SEM) is depicted across task blocks. Response accuracy significantly improved across task repetitions (Time effect). Lower panel: letter 2-back response latency [mean response latency (ms) ± 1 SEM] is depicted across task blocks.

Behavioral data demonstrated high task compliance across subjects and task blocks (mean correct: 94.8 ± 10.7%; mean response latency: 644 ± 171ms). One-way rmANOVA indicated a significant increase in mean accuracy across blocks [Time effect; *F*(6,90) = 2.39, *p* < 0.05, from 88.5% in Block 1 to 96.9% in Block 7; Figure 4; upper panel]. One-way rmANOVA indicated mean response latency non-significantly decreased across blocks [*F*(6,90) = 1.90, *p* = 0.09, from 697ms in Block 1 to 651ms in Block 7; Figure 4; lower panel].

### LCModel Fit Characteristics

**Figure 5:**
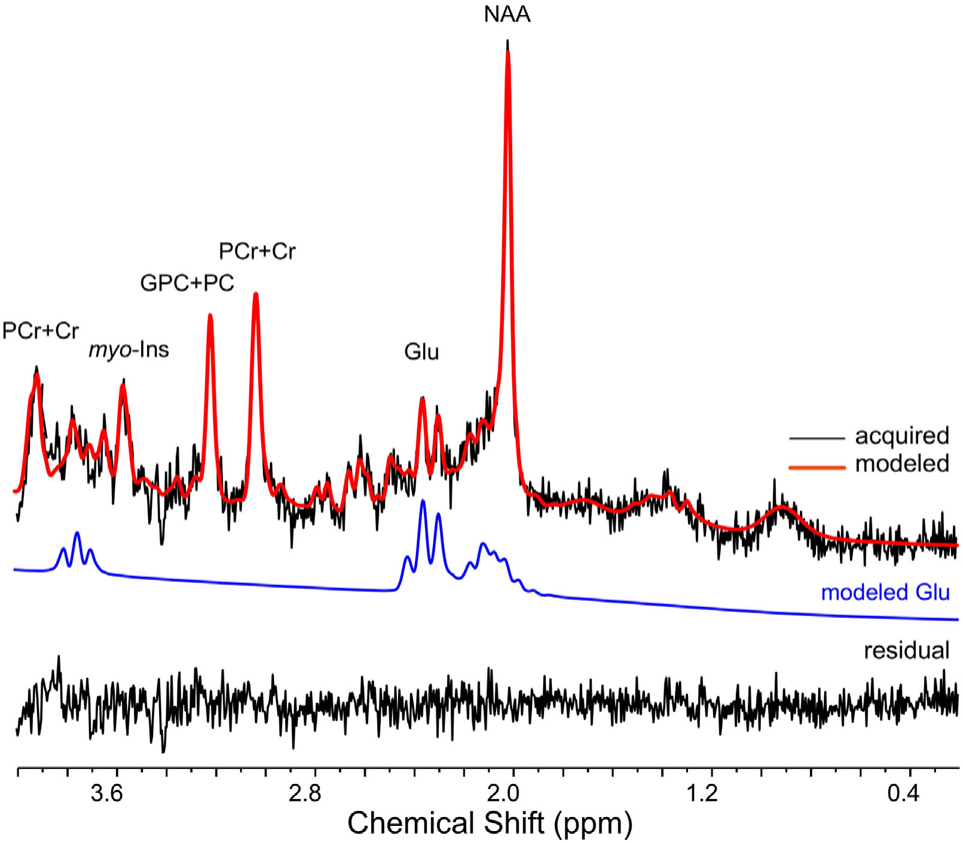
A representative ^1^H magnetic resonance spectroscopy spectrum is depicted (32s temporal resolution; 8 averages). The raw signal is depicted in black, while the LCModel fit is in red. The isolated glutamate (GLU) signal (blue line) and residual are presented below the spectrum. Chemical shift (ppm) is depicted below the spectrum. NAA, N-acetyl-aspartate; PCr+Cr phosphocreatine plus creatine; GPC+PC, glycerophosphocholine plus phosphocholine; *myo*-Ins, *myo*-Inositol.

LCModel fit characteristics are displayed in Table 1, and a representative spectrum is depicted in Figure 5. Exploratory analyses evaluated possible changes in LCModel fit characteristics as a function of task phase where 2-back-A and 2-back-B were considered separately. Two-way rmANOVAs indicated mean CRLB% of glutamate, FWHM, and SNR values did not differ across task blocks between 2-back and passive visual fixation (Table 1). These results indicate a non-significant BOLD effect on FWHM, and thus, linewidth broadening was not applied for subsequent analyses.

**Table 1:** LCModel Fit Characteristics by Task Phase.

LCModel fit characteristic	2-back-A	2-back-B	Interleaved visual fixation	Continuous visual fixation	*p* Values
Glutamate CRLB, %	6.8 ± 0.9	6.9 ± 0.9	6.8 ± 0.7	7.1 ± 0.9	>0.19
FWHM, Hz	4.9 ± 1.0	4.9 ± 1.0	5.0 ± 1.0	4.9 ± 0.9	>0.10
SNR	12.0 ± 1.9	12.1 ± 2.0	11.8 ± 1.7	11.7 ± 1.9	>0.13

*Mean ± 1 SD LCModel fit characteristics are described for each task phase. One-way repeated measures analyses of variance Task and Time X Task interaction effects contrasting 2-back (2-back-A and 2-back-B) vs. passive visual fixation (interleaved and continuous) conditions separately across task blocks indicated that LCModel fit characteristics were not systematically different between task phases (all *p* > 0.10)*.

*CRLB, Cramer Rao Lower Bound; FWHM, full width half-maximum in Hertz; Hz, Hertz; SNR, signal-to-noise ratio*.

### 2-Back Glutamate Modulation

**Figure 6:**
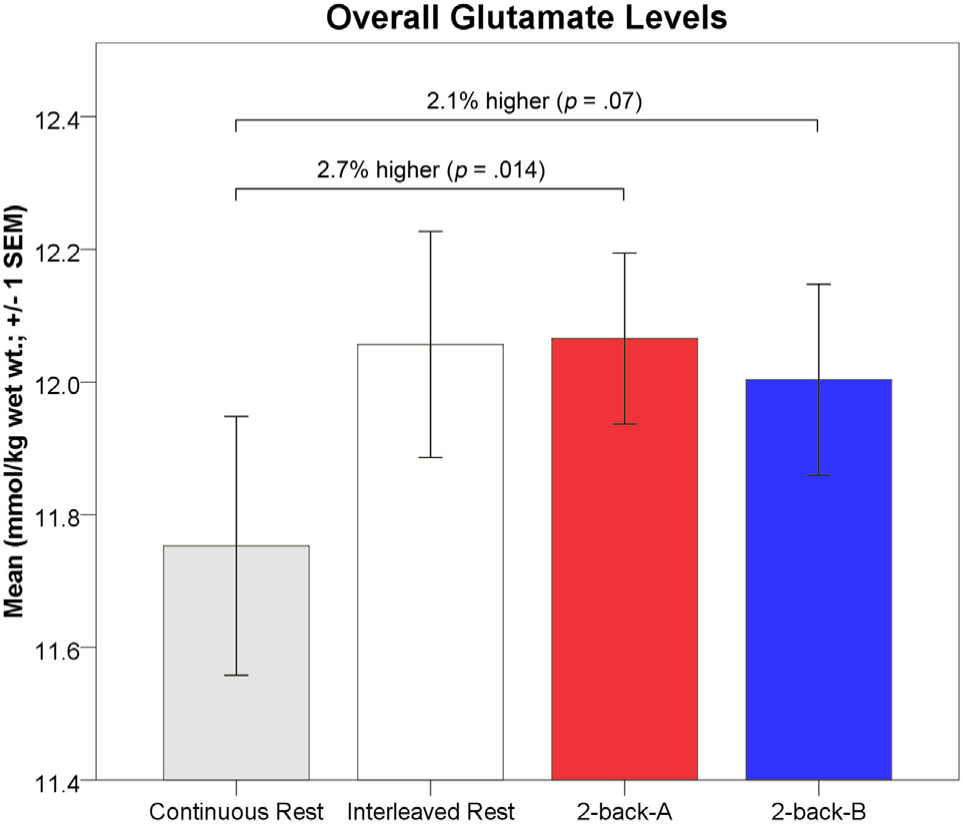
Absolute glutamate levels (mmol/kg wet wt. ± 1 SEM) for each task phase are depicted. Mean 2-back-A glutamate levels (red) were significantly higher (2.7%; 0.32 mmol/kg wet wt.) than continuous passive visual fixation glutamate levels (gray). Mean 2-back-B glutamate levels (blue) were non-significantly higher (2.1%; 0.25 mmol/kg wet wt.; *p* = 0.07) than continuous passive visual fixation glutamate levels (gray). Mean interleaved visual fixation glutamate levels (white) did not differ from 2-back levels.

As described above, glutamate levels during each 64s 2-back block were bisected into the first 32s, 2-back-A, and final 32s, 2-back-B, to match the 32s visual fixation measurements, such that the results were not biased by SNR and CRLB% of the glutamate fit. Overall, one-way rmANOVA indicated 2-back-A glutamate levels were significantly higher by 2.7% (0.32 mmol/kg wet wt.) than the continuous visual fixation levels [*F*(1,111) = 6.26, *p* = 0.014, partial η^2^ = 0.05; small-to-moderate effect size; 12.07 ± 0.85 vs. 11.75 ± 1.00 mmol/kg wet wt., respectively; Figure 6]. In contrast, 2-back-A glutamate levels did not differ from interleaved visual fixation levels (*p* = 0.92; 12.07 ± 0.85 vs. 12.06 ± 1.04 mmol/kg wet wt., respectively).

One-way rmANOVA indicated 2-back-B glutamate levels were non-significantly higher by 2.1% (0.25 mmol/kg wet wt.) than the continuous visual fixation levels [*F*(1,111) = 3.28, *p* = 0.07, partial η^2^ = 0.03; small-to-moderate effect size; 12.00 ± 0.98 vs. 11.75 ± 1.00 mmol/kg wet wt., respectively; Figure 6]. 2-back-B glutamate levels did not differ from interleaved visual fixation levels (*p* = 0.61; 12.00 ± 0.98 vs. 12.06 ± 1.04 mmol/kg wet wt., respectively).

### Dynamic Glutamate Levels

**Figure 7:**
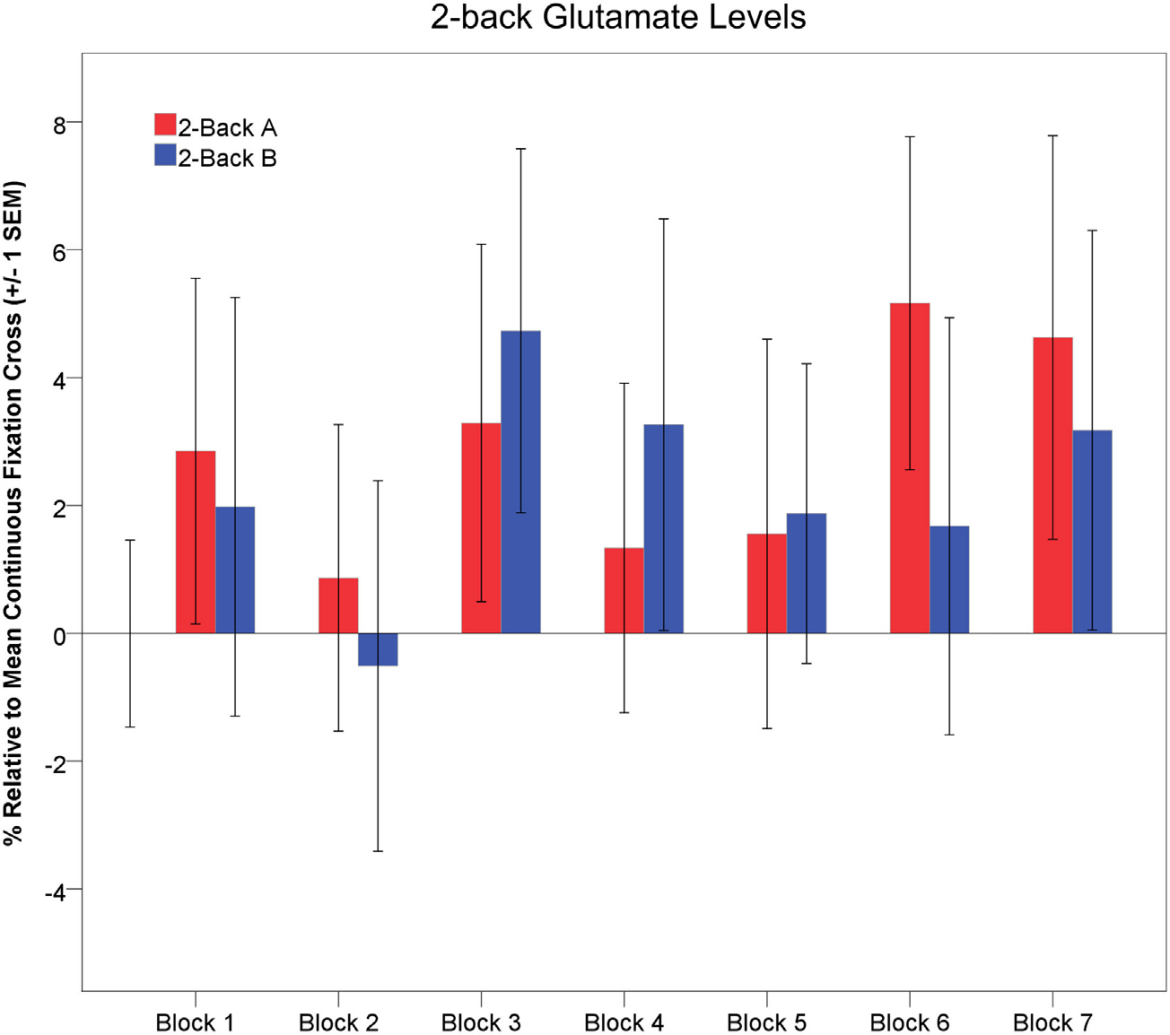
Glutamate levels (±1 SEM) are depicted across task blocks. Glutamate levels during 2-back-A (red) and 2-back-B (blue) are depicted as a percentage relative to the mean continuous visual fixation levels.

Exploratory one-way rmANOVAs indicated that glutamate levels did not change across task repetitions for either 2-back-A or 2-back-B (*p* > 0.35). However, glutamate levels significantly increased across task repetitions during the interleaved passive visual fixation [*F*(6,90) = 5.54, *p* < 0.001, partial η^2^ = 0.27; large effect size; not shown]. The temporal dynamics of 2-back glutamate levels relative to mean continuous visual fixation levels are depicted in Figure 7.

### Neurochemical Specificity

Metabolites other than glutamate were examined as a function of task demands: *myo*-Inositol, GPC+PC, PCr+Cr, and NAA. Exploratory two-way rmANOVAs indicated that metabolites (other than glutamate) did not significantly differ as a function of task phase relative to the continuous passive visual fixation.

### Exploratory Bivariate Correlations

Exploratory analyses examined bivariate correlations between glutamate levels and response accuracy. No significant zero-order correlations were observed (*p* > 0.15).

## Discussion

The goal of the present study was to detect changes in glutamate levels during performance of a well-established working memory task. Our findings indicated, for the first time in humans, that working memory processes increased *in vivo* glutamate levels in the dlPFC relative to the continuous passive visual fixation control condition. Throughout the remainder of the manuscript, we will interpret these findings, discuss study limitations, and describe future applications.

In the present study, ^1^H MRS spectra were continuously acquired from a small voxel with a volume of 4.5cm^3^ placed in the left dlPFC encompassing Brodmann areas 45 and 46 and analyzed at 32s temporal resolution with 8 averages per spectra. LCModel fit characteristics were used to confirm spectra quality. LCModel fit quality was consistent with previous ^1^H fMRS studies (31) and did not differ as a function task phase. CRLB% reflects the lower bound of the fitted parameters, and thus, it was critical to demonstrate that the quantification of glutamate was not biased by task phase (47). Relative to continuous passive visual fixation, *in vivo* left dlPFC glutamate levels were significantly higher by 2.7% (0.32mmol/kg wet wt.) during the first 32s and non-significantly higher by 2.1% (0.25mmol/kg wet wt.) during the final 32s of 2-back working memory task performance. The task related changes were specific to only glutamate, as changes in NAA, PCr+Cr, GPC+PC, and *myo*-Inositol were all non-significant. The magnitude of task-evoked glutamate modulation observed herein was consistent with prior ^1^H fMRS research (4–7, 10, 11). Finally, we found no significant bivariate correlations between response accuracy and glutamate levels, possibly due to a ceiling effect with mean response accuracy ~95%.

**Figure 8:**
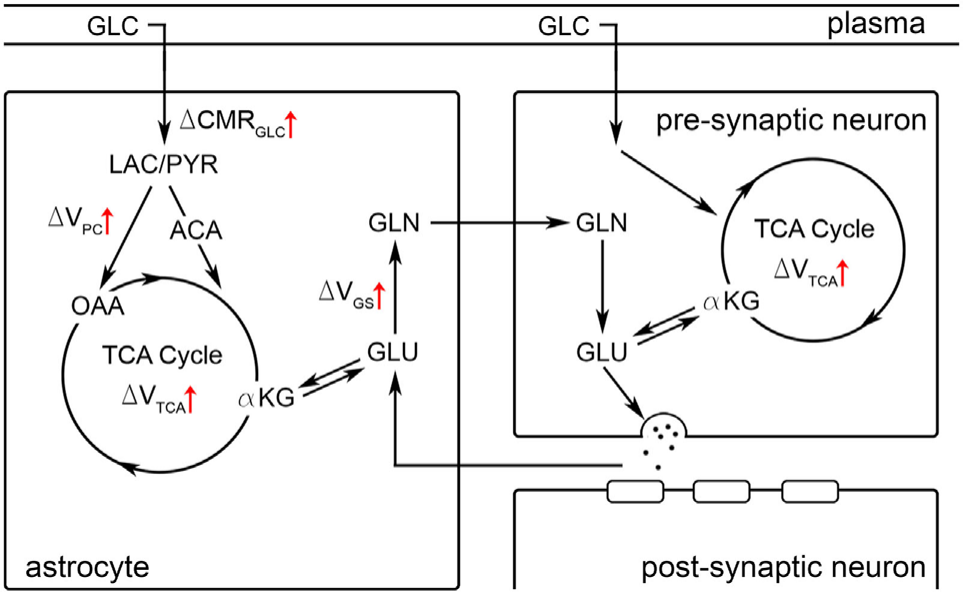
A schematic representation of the glutamatergic tripartite synapse is depicted. Black arrows illustrate relationships between molecular species. Red arrows illustrate enzymatic or metabolic reactions that are hypothesized to be upregulated during neural stimulation or cognitive task performance based on well-controlled preclinical studies which observed an increase in glutamate-glutamine cycling [e.g., Ref. (50)]. Net formation of glutamate during stimulation (i.e., as observed in ^1^H fMRS studies) is hypothesized to be associated with elevated glucose (GLC) extraction and metabolism (CMR_GLC_) (50). ACA, acetyl-CoA; αKG, α-ketoglutarate; CMR_GLC_, cerebral metabolic rate of glucose; GLC, glucose; GLN, glutamine; V_GS_, glutamine synthetase rate; GLU, glutamate; LAC, lactate; OAA, oxaloacetate; PYR, pyruvate; V_PC_, pyruvate carboxylation rate; V_TCA_, TCA cycle rate.

^1^H fMRS is a neuroimaging technique that facilitates direct measurement of dynamic neurochemistry levels. Seminal ^1^H fMRS research was conducted at high field, but technological advances have made detection of glutamate modulation possible at 3T (9, 15, 36). The ^1^H fMRS signal does not differentiate cell type, neuron vs. glia, or compartment, intracellular vs. extracellular, but is directly proportional to the concentration of molecules within the voxel. Thus, higher glutamate levels must reflect net formation of glutamate molecules. The putative neurobiological mechanism of net glutamate formation was clarified by extensive ^13^C MRS research which found a tight coupling between glutamate–glutamine neurotransmitter cycling rate and glucose metabolic rate (CMR_GLC_) in rodents across brain activity levels from isoelectricity to awake and supra-“basal” levels during stimulation (2, 20, 22, 23, 48–50). Thus, extant literature indicates that stimulation-induced increases in glutamate levels, as measured by ^1^H fMRS, reflect increased excitatory neurotransmission and oxidative metabolism. We suggest that working memory-related demands involving neural maintenance of letters during the delay periods drove an increase in excitatory neural activity in the dlPFC, which necessitated an increase in glucose utilization and oxidative metabolism. Behavioral data confirmed that all participants were actively engaged in the 2-back task and demonstrated high working memory proficiency: ~95% correct. Based on well-controlled preclinical work (50), the putative neurobiological pathways associated with net glutamate formation during working memory-related demand are illustrated in Figure 8. Prior studies have proposed increased enzymatic flux through several pathways, including pyruvate carboxylase and glutamate dehydrogenase (11, 21, 50) or the malate-aspartate shuttle (5, 51), but commentary about specific pathways is beyond the scope of our data. Multimodal studies indicate that stimulation-evoked glutamate modulation corresponded with other modalities indicating neural spiking activity, including BOLD activation (9, 10, 31) and gamma-band activity (8).

One prior ^1^H fMRS study investigated dynamic neurochemistry during working memory (34). Michels and colleagues (34) found GABA levels were significantly higher during the initial working memory task block. They interpreted the significant GABA modulation as indicative of increased metabolic activity and GABA-glutamate–glutamine cycling during working memory, as we do here (34). Interestingly, they found a significant decrease in GABA levels and response latency, but no change in response accuracy, across successive working memory task block repetitions (34). They interpreted these data to reflect improved working memory proficiency with repeated task performance. We found a similar learning, but different neurochemical, effect. Response latency non-significantly decreased and response accuracy significantly increased, across task repetitions. However, 2-back-A and 2-back-B glutamate levels were unchanged across task blocks (Figure 7). Thus, participants demonstrated enhanced working memory proficiency, but comparable glutamatergic response across task repetitions. There was some evidence of a more robust glutamatergic response early, as opposed to later, within each 64s 2-back block with nominally greater modulation during 2-back-A > 2-back-B, which may indicate the novelty of task onset contributed to the signal.

The ^1^H fMRS glutamate signal is a biomarker of the *relative* levels of excitatory neural activity between two states: stimulation vs. “basal” levels. Thus, isolation of task-evoked modulation necessitates accurate measurement of both states, which can be challenging, especially in the dlPFC. We selected the continuous visual fixation cross as our “non-task-active” behavioral paradigm based on prior research (36). In that study, we found glutamate levels exhibited less variability and lower levels, thus reflecting a better approximation of the “basal” state than other behavioral paradigms tested, such as eyes closed, flashing checkerboard and finger tapping (36). This is consistent with results herein that glutamate levels significantly increased across time during the interleaved passive visual fixation, reflecting less constrained behavior and a poor measurement of the “basal” state, while 2-back glutamate levels were unchanged across task blocks. This complicated our isolation of working memory-evoked glutamate modulation and illustrates the challenge of measuring dynamic neurochemistry in prefrontal brain regions. Prior ^1^H fMRS studies, conducted in motor and sensory cortices, demonstrated compelling stimulation-evoked change in glutamate levels in part because measurement of “basal” glutamate levels is simplified in those brain regions (4–7, 10, 11, 31). In the occipital lobe, the absence of visual stimulation is an ideal comparison for periods of visual stimulation (4). In contrast, the dlPFC is involved in numerous cognitive processes (52, 53), including fundamental processes, such as attentional control (54, 55). In theory, any cognitive process sub-served by the dlPFC could drive an increase in phasic neural activity and thus modulate glutamate levels. Indeed, Michels and colleagues found that dlPFC GABA levels exhibited high variability (non-significant linear regression) across time during four consecutive “rest” periods (34). This subtle complexity may have contributed to different analytic strategies and presentation styles. ^1^H fMRS studies that investigated cognitive task-related glutamate modulation often analyzed glutamate levels relative to a single baseline measurement (13, 15, 34), while ^1^H fMRS studies in sensory and motor regions typically depict dynamic glutamate levels across time using classical block designs as was used herein (4–7, 10, 11, 31). Future ^1^H fMRS studies that investigate neurochemical response during cognition should select their “non-task-active” behavioral paradigms with care, as accurate measurement of “basal” glutamate levels for comparison is essential to avoid Type II error (36).

Cognitive task-based^1^ H fMRS, as a neuroimaging approach, has numerous potential future applications and advantages over more widely used techniques (e.g., BOLD fMRI) (3). First, ^1^H fMRS facilitates direct *in vivo* measurement of dynamic neurochemistry, including the principal excitatory and inhibitory neurotransmitters (glutamate and GABA, respectively). Second, ^1^H fMRS is not susceptible to the neurovascular coupling effects associated with fMRI studies (3). As such, future ^1^H fMRS studies could examine populations not well-suited for fMRI studies such as elderly subjects, individuals with vascular diseases, or taking vasoactive medications. Third, the neurochemical specificity of this approach affords advantages for pharmacological challenge studies or investigation of neurobiological mechanisms. In particular, ^1^H fMRS is well-positioned for investigation of cortical excitability and the balance of excitatory and inhibitory drive, which is central to cognition and has been implicated in numerous psychiatric diagnoses (56).

### Alternative Explanations and Limitations

There are several alternative explanations for the present results. First, it is possible that *T_2_* relaxation of glutamate changed during 2-back task performance. The equation $S=S_{0}e^{-TE/T_{2}}$ describes the relationship between the initial signal (*S*_0_), the measured signal (*S*), *TE*, and *T_2_* relaxation (57). However, in order to explain the observed increase in glutamate concentration during 2-back, the *T_2_* relaxation of glutamate would need to increase ~20% assuming glutamate has *T_2_* value of 124ms at 3T in the dlPFC (58). Second, Type I error is possible. A small sample and winsorized distributions may increase likelihood of Type I error. However, results reported herein were hypothesized and consistent with published studies. In addition, the sample was unmedicated and healthy. Finally, we observed a small-to-moderate effect size using a rigorous experimental design.

This study has several limitations. First, as is the case with all single voxel MRS studies, this study was susceptible to partial volume effects. Three factors minimized the influence of partial volume effects on these findings: (1) voxel placement across subjects was highly reliable, (2) voxel tissue composition variability was low across subjects with gray and white matter CV% = 10.3 and 7.4%, respectively, and considered in the quantification of absolute glutamate concentration, and (3) only within-subject analyses across conditions were considered. A second limitation was our inability to reliably quantify other relevant molecules such as GABA, glutamine, aspartate, or lactate. A third limitation was voxel placement based on a BOLD fMRI meta-analysis (35) and not from the measured BOLD activation of the individual subjects. Fourth, we did not measure neurochemistry from a “control” voxel location. Finally, it is important to note that other ^1^H fMRS studies observed significant modulation of metabolites other than glutamate (17). As such, we chose not to report glutamate levels as a ratio relative to other metabolites such as PCr+Cr or NAA, because any modulation of those metabolites in time would confound interpretation. Indeed, prior ^1^H fMRS studies found significant NAA modulation as a function of task stimulation (59–61) and others found non-significant, but notable, PCr+Cr modulation (10, 11).

### Conclusion

The findings presented herein demonstrated, for the first time in humans, that working memory task performance modulated *in vivo* dlPFC glutamate levels in healthy adult volunteers. Results from this study were consistent with *a priori* hypotheses and prior research across modalities including fMRI, ^1^H fMRS, and electrophysiology. We interpret elevated glutamate levels during working memory task performance to reflect increased metabolic activity and excitatory neurotransmission driven by working memory-related demands. Future research studies are needed to replicate these effects and investigate possible “dose-response” relationships with glutamate modulation as a function of working memory cognitive load.

## Ethics Statement

This study was carried out in accordance with the recommendations of Wayne State University Institutional Review Board with written informed consent from all subjects. All subjects gave written informed consent in accordance with the Declaration of Helsinki. The protocol was approved by the Wayne State University Institutional Review Board.

## Author Contributions

EW authored the manuscript and developed the figures. EW and JS developed the experimental task, analyzed the data, and edited the manuscript. CA assisted with data analyses and edited the manuscript. DK operated the MRI scanner and assisted with data collection. VD assisted with experimental task development and edited the manuscript. All authors have read and approved of this manuscript.

## Conflict of Interest Statement

All authors declare no conflict of interest with respect to the conduct or content of this work.
